# High resolution thermal remote sensing and the limits of species’ tolerance

**DOI:** 10.7717/peerj.13911

**Published:** 2022-09-28

**Authors:** Gabrielle Ednie, Jeremy T. Kerr

**Affiliations:** Department of Biology, University of Ottawa, Ottawa, Ontario, Canada

**Keywords:** UAV, UAS, Thermal map, Thermal imagery, High-resolution remote sensing, Climate change, Conservation biology, Microclimate, Organismal climatology, Thermal positioning

## Abstract

Extinction risks for many insect species, particularly across very broad spatial extents, have been linked to the growing frequency and severity of temperatures that exceed the boundaries of their realized niches. Measurement and mitigation of such impacts is hindered by the availability of high-resolution measurements of species-specific severity of extreme weather, especially temperature. While techniques enabling interpolation of broad-scale remote sensing metrics are vital for such efforts, direct remote sensing measurements of thermal conditions could improve habitat management by providing detailed insights that interpolative approaches cannot. Advances in unmanned aerial vehicle (UAV) technology have created opportunities to better evaluate the role of microclimates in local species extinctions. Here, we develop a method to create high-resolution maps of microclimates using UAV and thermal imaging technology that use species’ realized niche boundaries to assess potential effects of severity of extreme temperatures. We generated air temperature maps (5 cm resolution) and canopy height maps (1 cm resolution) for 15 sites in a rare alvar ecosystem in eastern Ontario. We validated these remote sensing observations against independent, *in situ* temperature observations using iButtons. Temperature observations were accurate and related to physical heterogeneity in alvar habitats. We converted temperature measures into estimates of proximity of thermal niche boundaries for three butterfly species found during field surveys. This is the first time that this method has been applied to high resolution remote sensing observations and offers potential to assess the availability and adequacy of microclimates within habitats at resolutions relevant for conservation management.

## Introduction

Climate change exposes species to abiotic conditions that may exceed their tolerances (Kerr et al., 2015; Urban et al., 2016), leading to growing frequencies and severities of extreme weather events (Harris et al., 2018; Kerr, 2020). Such changes contribute to the declines of many species (Riddell et al., 2021; Soroye, Newbold & Kerr, 2020). Over broad geographical areas, such extreme events are increasing extinction risks for populations of key pollinator species (Soroye, Newbold & Kerr, 2020) and vertebrates at global extents (Williams & Newbold, 2021). Distinguishing between effects of “press” events (*e.g.*, shifts in average climatic conditions that can progressively change the suitability of an environment for particular species) *vs.* “pulse” events (*e.g.*, short duration extreme weather that can cause population decline; Harris et al., 2018), temperature extremes (“pulse” events) in particular have been linked to changes in species colonization-extinction dynamics, contributing to declines for many species across broad geographical areas. Translating broad-scale models to direct local measurements that assess species’ exposures to extreme temperature, relative to their individual tolerances, could improve habitat management and species’ conservation prospects.

Microclimate refugia are areas where species can find shelter from extreme weather (Rull, 2009). The size of these refugia depends on the body size and niche boundaries of each species (Keppel et al., 2012). Species distribution models (SDMs) are often used to forecast impacts of climate change on species’ ranges (Algar et al., 2009; Kharouba, Algar & Kerr, 2009; Porfirio et al., 2014). However, such methods rely heavily on long term climate data and are more appropriate for use at large biogeographical extents (Anderson & Gaston, 2013; Ashcroft, 2010; Potter, Woods & Pincebourde, 2013). Species experience temperatures at very localized spatial extents (Suggitt et al., 2011). While some studies have measured microclimatic variation of complex local landscapes at scales relevant to the movement and habitat use of individual organisms, fewer studies have assessed this microclimatic variability relative to individual species’ thermal boundaries comprehensively throughout habitats (Milling et al., 2018; Pincebourde et al., 2016; Rebaudo, Faye & Dangles, 2016; Slavich et al., 2014; Suggitt et al., 2011; Suggitt et al., 2018). A key challenge is that many habitats exhibit considerable thermal heterogeneity (*e.g.*, Milling et al., 2018), which can enable species to find shelter from short duration temperature extremes (Suggitt et al., 2011; Suggitt et al., 2018). Techniques to measure microclimate heterogeneity relative to the limits of species’ tolerances are essential for predicting extinction risks of small-bodied species (Pincebourde et al., 2016; Potter, Woods & Pincebourde, 2013; Rebaudo, Faye & Dangles, 2016; Suggitt et al., 2018), but are likely to require emerging remote sensing technologies (Zellweger et al., 2019).

Unmanned aerial vehicles (UAVs, or drones) show considerable promise in ecological research (Christie et al., 2016; Duffy et al., 2021; Zellweger et al., 2019). The availability of powerful, lightweight sensors, including thermal, multispectral, visible light, and LiDAR, create opportunities to translate broad-scale models to particular habitats, which could help predict movements or presences of individual species within habitats (Anderson & Gaston, 2013; Duffy et al., 2021; Zellweger et al., 2019). Satellite thermal infrared (TIR) imagery and topographical data have been used in broad-scale ecological models (Zellweger et al., 2019). However, most satellite TIR imagery resolution is too coarse to detect and measure microclimates directly, particularly for small-bodied organisms, which may limit their application to air and soil microclimatic temperature measurements in some cases (Helmuth et al., 2010; Anderson & Gaston, 2013; Zellweger et al., 2019). Radiometric thermal cameras mounted on UAVs provide measurements at very high resolutions that can complement broader-scale remote sensing measurements of temperature (Anderson & Gaston, 2013; Brenner et al., 2018; Byerlay et al., 2020; Maes, Huete & Steppe, 2017; Messina & Modica, 2020; Milling et al., 2018). Prior to the onset of UAV and thermal camera technologies, microclimate studies required temperature loggers, such as iButtons (George, Thompson & Faaborg, 2015; Holden et al., 2011). Such loggers are vital for calibrating and validating thermal remote sensing observations, but remote sensing provides unique advantages in terms of synoptic environmental measurement that greatly expands the reach of *in situ* ecological measurement (George, Thompson & Faaborg, 2015; Holden et al., 2011; Kerr & Ostrovsky, 2003).

The thermal limits of each species could predict the response of small-bodied species to climate change (Sunday, Bates & Dulvy, 2012). There is mounting evidence of species altering their historical range in response to habitats exceeding their thermal limitations (Hufnagel & Kocsis, 2011; Soroye, Newbold & Kerr, 2020; Williams & Newbold, 2021). When temperatures exceed a species’ thermal tolerances, their fecundity and survival declines because they must expend energy on behavioural or physiological thermoregulation rather than resource gathering or reproduction (*e.g.*, Buckley, Schoville & Williams, 2021). The newly-developed and tested Thermal Position Index (TPI; Kerr, 2020; Soroye, Newbold & Kerr, 2020; Williams & Newbold, 2021) relates species’ realized thermal niches to their extinction-colonization dynamics. This method measures thermal tolerances using historical observations of air temperatures in areas where species have successfully persisted over time. Species’ upper thermal limits evolve slowly, so adaptation rates are likely to be insufficient to permit many species to tolerate rapid warming (Araújo et al., 2013; Bennett et al., 2021).

This paper proposes a new methodological framework to measure landscape-scale microclimatic profiles with UAV and thermal infrared imaging technology, and illustrates their use in a practical conservation setting. We simultaneously outline a method of translating the Thermal Positioning Index, previously validated at global scales, to microclimatic applications. This framework includes five steps: data collection, assessment of species’ thermal limits, map building, mapping of thermal conditions relative to species’ measured tolerances, and interpretation (*sensu* Faye et al., 2016). We present examples of how individual species’ tolerances can be linked to remotely sensed thermal data to describe habitat suitability for three butterfly species: *Hesperia sassacus* (Indian skipper), *Speyeria aphrodite* (Aphrodite fritillary), and *Coenonympha tullia* (Common ringlet).

## Materials & Methods

### Step 1—Data collection

**Study Site.** Field sites were located in Burnt Lands Provincial Park situated 30 km west of Ottawa, ON, which hosts an alvar ecosystem interspersed with mostly coniferous tree stands. Fifteen sites of varying sizes separated by a minimum of 20 m of forested area were selected (Gordon & Kerr, 2022). All sites consisted of open areas and clearings. Only two were not surrounded by trees. Research and UAV use permits were provided by Ontario Parks. Recognized as rare and imperiled ecosystems by the Nature Conservancy of Canada, alvars are characterized by open and barren areas with little to no soil, and often host rare species (NCC, 2020). During summer, these landscapes can experience highly localized extreme heat in areas with exposed limestone, while vegetated areas nearby might have considerably lower temperatures. The spatial variability in these thermal conditions has not previously been measured.

**UAV and Sensor.** A DJI Matrice 300 quadcopter with real-time kinetic (RTK) positioning was deployed. This drone carried a Zenmuse XT2 dual sensor with thermal (13 mm focal length; 640 × 512 image capture) and visual (eight mm focal length, 12 megapixel resolution) imaging capabilities (DJI Inc., Shenzhen, China). A RTK base station was deployed in the field, which increased the positioning accuracy of the UAV by providing real-time differential corrections, and eliminated the need for ground control points. The quadcopter was equipped with the DJI pilot program, which included a mission function allowing execution of automated flight and camera control sequences (DJI, 2021). Imaging was acquired during missions programmed in the DJI pilot program using satellite imagery. The thermal camera captured images in the thermal infrared (TIR) spectral range in the radiometric-jpeg (R-JPEG) format. Each pixel was embedded with temperature data. The in-camera emissivity value was set to one for TIR images and adjusted in the GIS workflow step outlined below. The visual camera captured images in the red, green, and blue spectral bands (RGB). Both cameras captured images simultaneously. Every image was geotagged with the RTK-corrected GPS coordinates.

**Flight Plan.** Image acquisition flight plans were programmed with a 90% image overlap on all sides to optimize mapping accuracy, as recommended by the Pix4DMapper software used in the mapping step (Pix4D SA, Lausanne, Switzerland; Pix4D, 2021). The UAV was programmed to capture images at 1 s intervals and fly at a constant 2.5 m/s speed to maximize survey area, given a 37-minute battery life limitation, while minimizing motion blur. All missions were performed at 37 m altitude to achieve five cm thermal imaging resolution and one cm RGB imaging resolution. All flights were restricted to days above 15 °C with <50% cloud cover between 10:30 am and 3:30 pm to ensure the accuracy and comparability of the thermal imagery gathered (Dai, Trenberth & Karl, 1999). Cloud cover alters TIR-based temperature measurement, so all flight missions were paused during cloudy periods and resumed after they cleared. Missions were aborted if conditions remained cloudy. Butterfly surveys were conducted in parallel to our UAV surveys. While the data was not used in this paper, the butterfly monitoring methodology’s temporal and temperature requirements (*i.e.,* between 10:45 am and 3:45 pm, and over 13 °C) had to be respected for the drone surveys as well (Pollard, 1977). As the method was designed for British summer conditions, mild liberties were taken with the methodology (*i.e.,* earlier start but higher temperature requirement).

**Figure 1:**
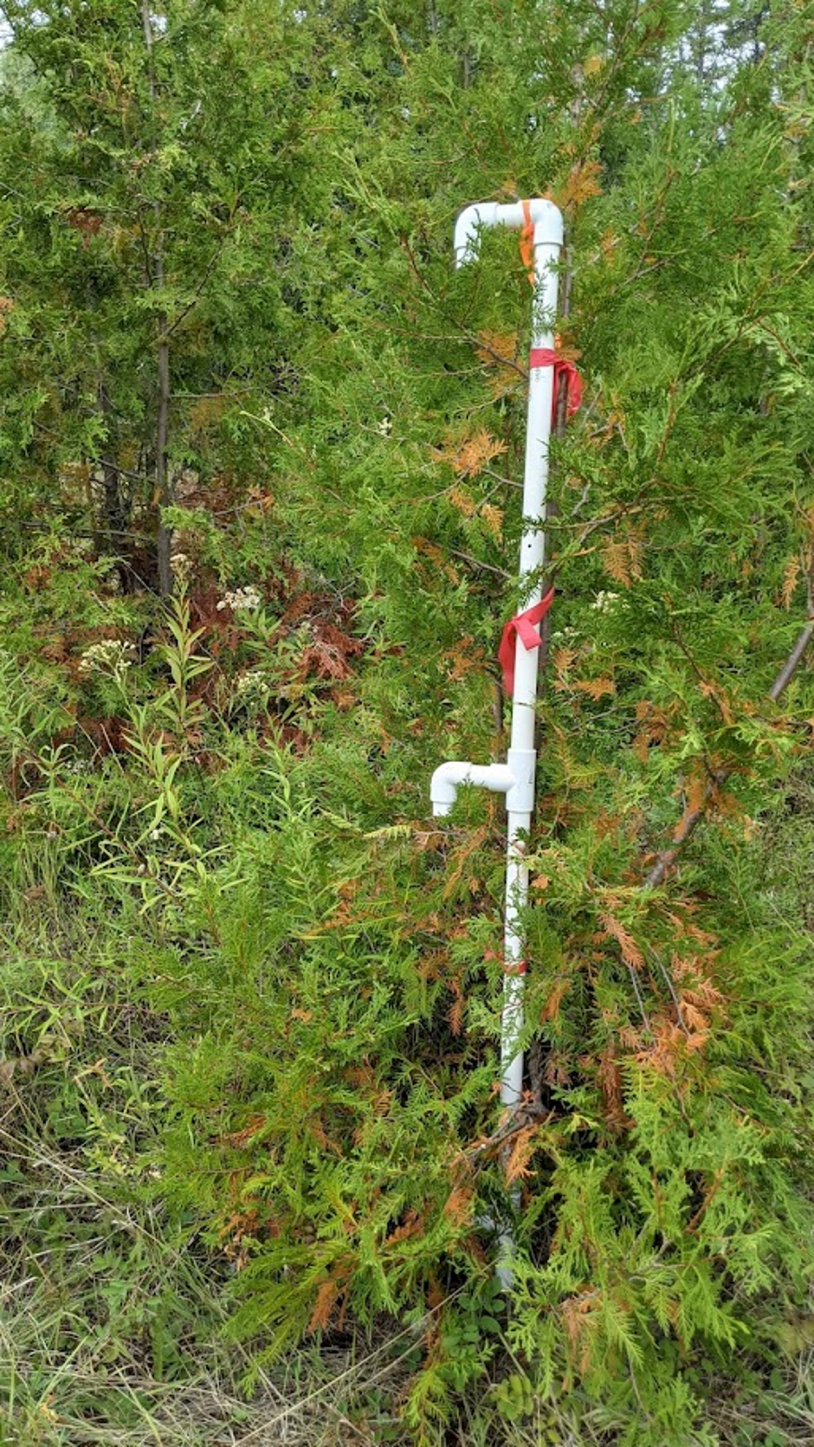
Image of a PVC pole containing iButtons at 0.05 m, 0.75 m, and 1.5 m deployed in the field. Photo credit: Gabrielle Ednie.

**In situ Temperature Measurements.** To calibrate temperature readings captured by the thermal imaging, temperature loggers were placed *in situ*. At each site, an iButton (DS1922L-F5#, Maxim, Dallas, USA; accuracy: ±0.5 °C) coated in clear plastic (Roznik & Alford, 2012) was placed on the ground approximately 1 meter into the tree line in full shade near each site’s access point for convenience. The plastic coating provided a waterproofing barrier (Plasti Dip, Blaine, MN, USA) for the iButton but is not expected to significantly affect the air temperature readings in the shade (Roznik & Alford, 2012). These temperature loggers (hereafter referred to as ground loggers) were assumed to measure near surface air temperature as tree shade blocked most solar radiation and acted as solar shields (Gies et al., 2007). Statistical verifications were made to support this assumption. Three ground loggers were lost in the field, likely due to wildlife interference. At three sites, three poles each containing three uncoated iButtons at 0.05 m, 0.75 m, and 1.5 m (total of 27 iButtons) were placed to record air temperature variations at different heights (Fig. 1). These poles were constructed out of white PVC pipes (Mittra, van Etten & Franco, 2013). The three sites were chosen for their variation in dominant surface type (limestone, grass, and mix of both). Temperature loggers (hereafter referred to as pole loggers) were positioned on the tip of each protrusion and rest on wire mesh to allow ventilation. Additional holes were drilled along the main pole and on each protrusion to allow better ventilation. These temperature loggers were used to model the relationship between UAV captured remotely sensed soil surface temperatures and air temperatures as air temperature is the metric adult lepidopterans, our study group, are most exposed to. Every iButton was programmed to record temperature at 30-minute intervals and was placed in the field only to be retrieved at the end of the field season. Air temperature was also measured before every UAV mission in a shaded area using a handheld HT-86 humidity meter (Wal Front, USA; accuracy: ±0.5 °C, ±3% RH).

### Step 2—Generating thermal limits

We extracted data on the five hottest and coldest locations in the ranges of butterfly species that were detected in our study sites based on a historical air temperature dataset (Harris et al., 2014). As in Soroye, Newbold & Kerr (2020), we used a baseline observation period to estimate thermal limits. Only occurrences between 1901 and 1975 were considered when estimating species’ upper and lower thermal limits. Climate change has accelerated rapidly after that baseline period. By using the location-month combinations, only the months where a species observation had occurred were considered to extract monthly maximum and minimum air temperatures. Therefore, the summer months of the overwintering sites would not be considered when extracting thermal limits. Location-month combinations were used in lieu of location-day combinations due to lack of historical daily temperature data. These values have previously been shown to be informative with respect to insect and other species’ vulnerabilities to changing frequencies of extreme weather (Outhwaite, McCann & Newbold, 2022; Soroye, Newbold & Kerr, 2020; Williams & Newbold, 2021). Historical air temperatures were obtained from the Climate Research Unit dataset (Harris et al., 2014). Lepidoptera occurrence information was extracted from the eButterfly citizen science program (Prudic et al., 2017) and from longer term butterfly observations assembled through the activities of systematists and biological surveyors (Soroye, Ahmed & Kerr, 2018). Each species observation is traceable to a curated museum specimen or to a submitted observation that has been approved by a team of butterfly experts.

### Step 3—Mapping

A total of 30 drone surveys were conducted from May 17 to August 26, 2021. One survey was discarded due to a brief malfunction with the RTK base station, which caused georeferencing discrepancies. As a result, every raster output was produced 29 times for each of the drone surveys. Raw TIR and RGB images collected in the field were used to generate TIR, RGB, digital surface model (DSM), and digital terrain model (DTM) orthomosaics (*i.e.,* a georeferenced aerial image geometrically corrected; Faye et al., 2016) using the Pix4Dmapper software. The software used the embedded GPS information in each image and detected characteristic objects in the images to generate tie points and create densified point clouds. These clouds were then used to blend overlapping images and create an orthomosaic (hereafter referred to as map) with the original pixel information still intact. For each of the 29 surveys, one map of each type (TIR, RGB, DSM, DTM) was created. TIR maps had an approximate resolution of five cm/pixel, while RGB, DSM, and DTM maps had an approximate resolution of one cm/pixel.

### Step 4—GIS Processing

**Classified Surface Type Map.** The RGB maps were then imported into ArcGIS Pro software (ArcGIS Pro. Version 2.5; Esri, Redlands, CA, USA). Thermal cameras estimate soil temperature by measuring the amount of infrared energy being reflected from the ground (Madding, 1999). However, each surface reflects, absorbs, and emits re-radiated light differently (*i.e.,* emissivity). To better estimate soil surface temperature, correcting for surface emissivity is essential (Madding, 1999). To correct the remotely sensed soil surface temperature TIR maps for emissivity, each RGB map had to be classified by surface type (Becker, 1987; Faye et al., 2016). This was accomplished using the *Classification Wizard* tool. The following surface types were included in the classification schema: debris, forest, grass, tall grass, limestone, shrub, soil, water, and wood. An object-based classification type was used using a supervised classification method. In each RGB map, approximately 25% of each surface type was identified using the *Segment Picker* tool. This process generated a classified raster with each pixel identified as the appropriate surface type. These maps were validated by matching ground truth data about major landscape features to the land cover classification.

**Emissivity Map.** To create emissivity rasters, an “Emissivity” field was added to the classified maps’ attribute tables. The emissivity values were added manually based on a literature review (Table 1). Objects identified as debris were given an emissivity value of 1 as their composition was not always known. Each map was then resampled to match the pixel size of the classified maps to the pixel size of the thermal maps. The emissivity values were extracted into a new raster and turned into floating point rasters to ensure the emissivity map was in the same raster format as the TIR maps.

**Emissivity-Corrected Remotely Sensed Soil Surface Temperature Map.** Emissivity-corrected soil surface temperature maps were created by multiplying the TIR maps with the emissivity maps. The difference in focal length between the TIR and visual cameras caused occasional misalignments between the RGB and TIR maps. As such, the emissivity and TIR maps were first manually aligned.

**Modelling Air Temperature**. To transform the remotely sensed soil surface temperature maps into air temperature maps, we modelled the relationship between the air temperatures (ground and pole logger data) and soil surface temperatures (emissivity-corrected remotely sensed soil surface temperature maps) at a given position. We ensured air temperature data of different logger heights was not statistically different before performing the model. The mean temperature in a 30 cm radius around the iButton locations were extracted and used as soil surface temperature proxy on the corrected soil surface temperature maps. The iButton temperature recorded nearest the time of the UAV survey was used as air temperature. A simple linear regression model with 76 data points was constructed in R to relate air temperature to remotely sensed soil surface temperature used here as the independent variable (R Core Team, 2019). Although air temperature data recorded *via* iButtons was considered to represent “true” temperatures, it was used as the dependent variable. This allowed for an easier calibration of remotely sensed soil surface temperatures into air temperatures. As this regression model was statistically strong, we used the model slope’s equation to calibrate drone-based ground temperature maps into air temperature measurements.

**Table 1:** Emissivity values used for different land surfaces.

**Surface type**	**Emissivity**	**Source**
Forest	0.99	Sobrino, Jiménez-Muñoz & Paolini (2004)
Grass	0.98	Labed & Stoll (1991)
Tall Grass	0.994	Labed & Stoll (1991)
Limestone	0.95	Mineo & Pappalardo (2021)
Shrub	0.986	Van de Griend & Owe (1993)
Soil	0.95	Nichol (2009)
Water	0.995	Qin et al. (2006)
Wood	0.97	Pitarma, Crisóstomo & Jorge (2016)

Soil surface temperatures differ greatly between shaded and open areas, primarily due to solar radiation. The effects of radiation on organismal body temperatures are complex, depending on factors such as behaviour, body size, and coloration (Stelbrink et al., 2019; Stevenson, 1985). To avoid systemic biases due to variability in solar radiation, we limited UAV operation to cloud-free times around mid-day and treated radiation as a constant in our subsequent modelling (Dai, Trenberth & Karl, 1999). We opted to measure *in situ* air temperature in the shade, as convention dictates when measuring ambient air temperature, to find a single generalized conversion factor for soil surface to air temperature over our study sites. This methodology was developed to require minimal microhabitat temperature modelling. Therefore, pole loggers were placed in sites with varying surface types to account for the landscape variability, and data were pooled together to generalize the model across the study sites. The resulting air temperature model remained statistically strong and facilitates its reproducibility in different ecosystems. A main objective of our study was to adapt a verified global index of species vulnerability to microclimatic scales. A big component of this index uses historical air temperature data captured from meteorological stations to estimate species thermal niche boundary. As such, air temperature measurements needed to be used to generate the thermal positioning index and overheating index of each species.

**Figure 2:**
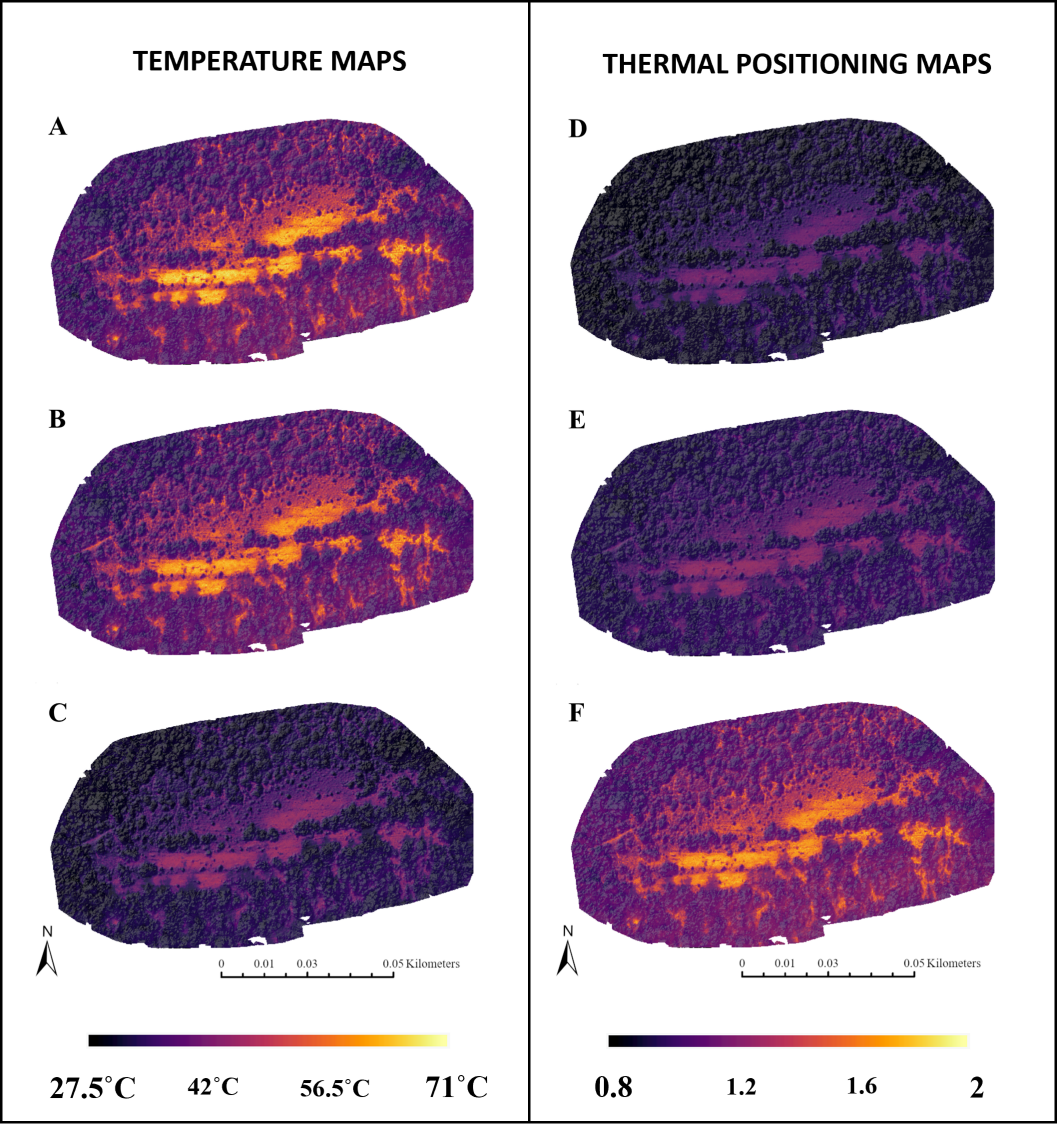
Three temperature maps and three thermal positioning maps of a survey completed on August 3rd, 2021. The maps were rendered slightly transparent and overlaid on a shaded relief map of its canopy height map to depict topographic variation also. The maps shown are as follows: (A) raw remote sensing temperature map, (B) emissivity-corrected remote sensing map, (C) air temperature map, (D) *C. tullia* thermal positioning map, (E) *S. aphrodite* thermal positioning map, and (F) *H. sassacus* thermal positioning map. For the thermal positioning maps, a value of 1 represents a pixel with a temperature value equal to the upper thermal limit. Values exceeding 1 represent pixels with temperature readings greater than the upper thermal limit of the species.

**Air Temperature Map**. Air temperature maps were extrapolated from the emissivity-corrected remotely sensed soil surface temperature maps using the aforementioned air temperature model equation (Fig. 2). These maps were used in step 5 (see below) as they better represent the thermal conditions experienced by animals and airborne insects such as *Lepidoptera*.

**Thermal Positioning Map**. Thermal positioning maps were generated using the historical thermal limits of the study species (*H. sassacus*, *S. aphrodite*, and *C. tullia*) and the air temperature maps. Thermal positioning maps estimate a species’ proximity to its thermal limits in every pixel. These maps were estimated as

\begin{eqnarray*}P= \frac{{N}_{m}-{N}_{Smin}}{{N}_{Smax}-{N}_{Smin}} , \end{eqnarray*}

developed by Soroye, Newbold, and Kerr (2020), where P is the species’ thermal position at a given location or pixel, *N*_m_ isthe air temperature of a given pixel in the air temperature map, *N*_Smax_ is the species’ upper thermal limit, and *N*_Smin_ is the species’ lower thermal limit (Fig. 2). This index has previously been shown to predict extinction risk among bumblebees, aspects of population dynamics among mammals, and insect declines more generally (Kerr, 2020; Outhwaite, McCann & Newbold, 2022; Soroye, Newbold & Kerr, 2020; Williams & Newbold, 2021; Williams et al., 2022). A value of one represents a pixel with a temperature value equal to the upper thermal limit. Values exceeding one represent pixels with temperature readings greater than the upper thermal limit of the species.

**Canopy Height Map**. Canopy height maps were generated by subtracting the digital terrain maps from the digital surface maps. Terrain maps represent ground topography, while surface maps represent an elevation map of both natural and artificial features in addition to ground topography. The resulting canopy height maps represent the height of the natural and artificial features.

### Step 5—Ecological indices

**Overheating Index.** The overheating index was used as a landscape-scale relative heat indicator. It was calculated as the proportion of pixels within the UAV temperature measurement area where that species’ thermal position was ≥ 1. For thermal position index, such values indicate that temperatures exceed the boundaries of that species’ upper thermal limits.

**Foliage Height Diversity.** Foliage height diversity represents the canopy height diversity and is used as a landscape heterogeneity index (MacArthur & MacArthur, 1961). We classified canopy height maps to the nearest 0.5 m interval and calculated the inverse Simpson index to assess this aspect of heterogeneity.

**Figure 3:**
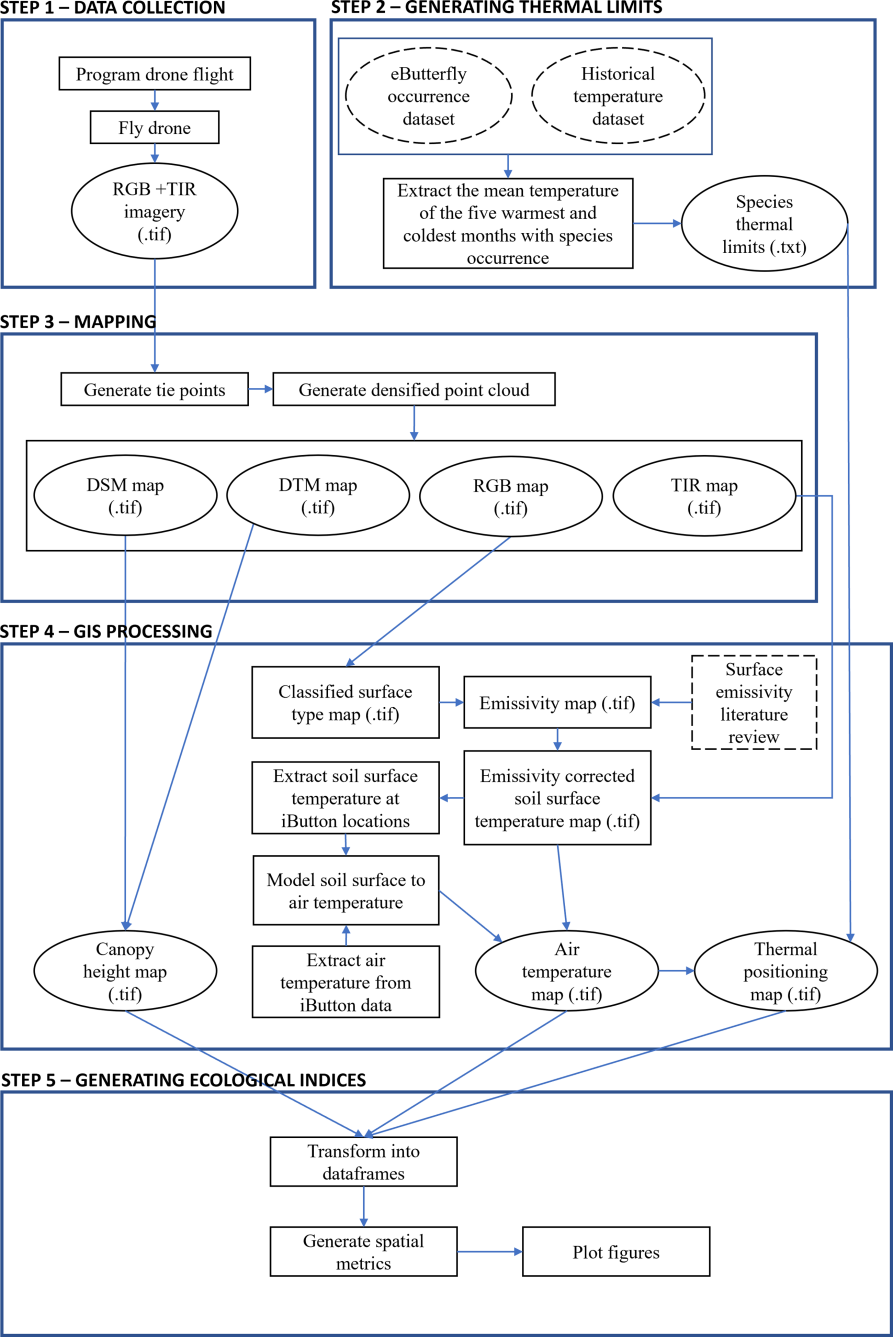
Flowchart of the proposed methodological framework. Dashed lines represent data from outside sources. Rectangular shapes represent intermediate outputs and steps. Oval shapes represent primary outputs from each step. All soil surface temperatures were remotely sensed.

**Thermal Diversity.** Lastly, we assessed thermal diversity in a similar manner to foliage height diversity. First, we classified temperature data according to the nearest 0.5 °C temperature interval, and then calculated the standardized inverse Simpson index for each site (Faye et al., 2016; Fig. 3).

### Study species

Butterflies were used as focal species. Butterflies are useful model organisms for small-scale climate change research (Beirão & Cardoso, 2020). Due to their small size and dependence on temperature to regulate body heat, insects are considered good model organisms to predict species response to climate change (Beirão & Cardoso, 2020; Wilson & Maclean, 2011). However, few insect species have detailed contemporary and historical datasets like *Lepidoptera* (Wilson & Maclean, 2011). As a result, the impact of climate change on butterflies has been well documented (Beirão & Cardoso, 2020; Hufnagel & Kocsis, 2011; Mattila et al., 2011; Wilson & Maclean, 2011). We assessed thermal position indices for three butterfly species (*Hesperia sassacus*, *Speyeria aphrodite*, and *Coenoympha tullia*) that account for microclimatic variation at scales relevant to these species’ individual movements and thermoregulation. These species were selected for their variation in body size, taxonomy, and thermal tolerance (5.40 °C to 28.56 °C, −14.78 °C to 32.37 °C, and −12.65 °C to 36.04 °C respectively). Each species was observed during transect based butterfly surveys. Beyond confirming the presence of our case study species at our study sites, results from these surveys are outside the scope of this paper.

## Results

**Figure 4:**
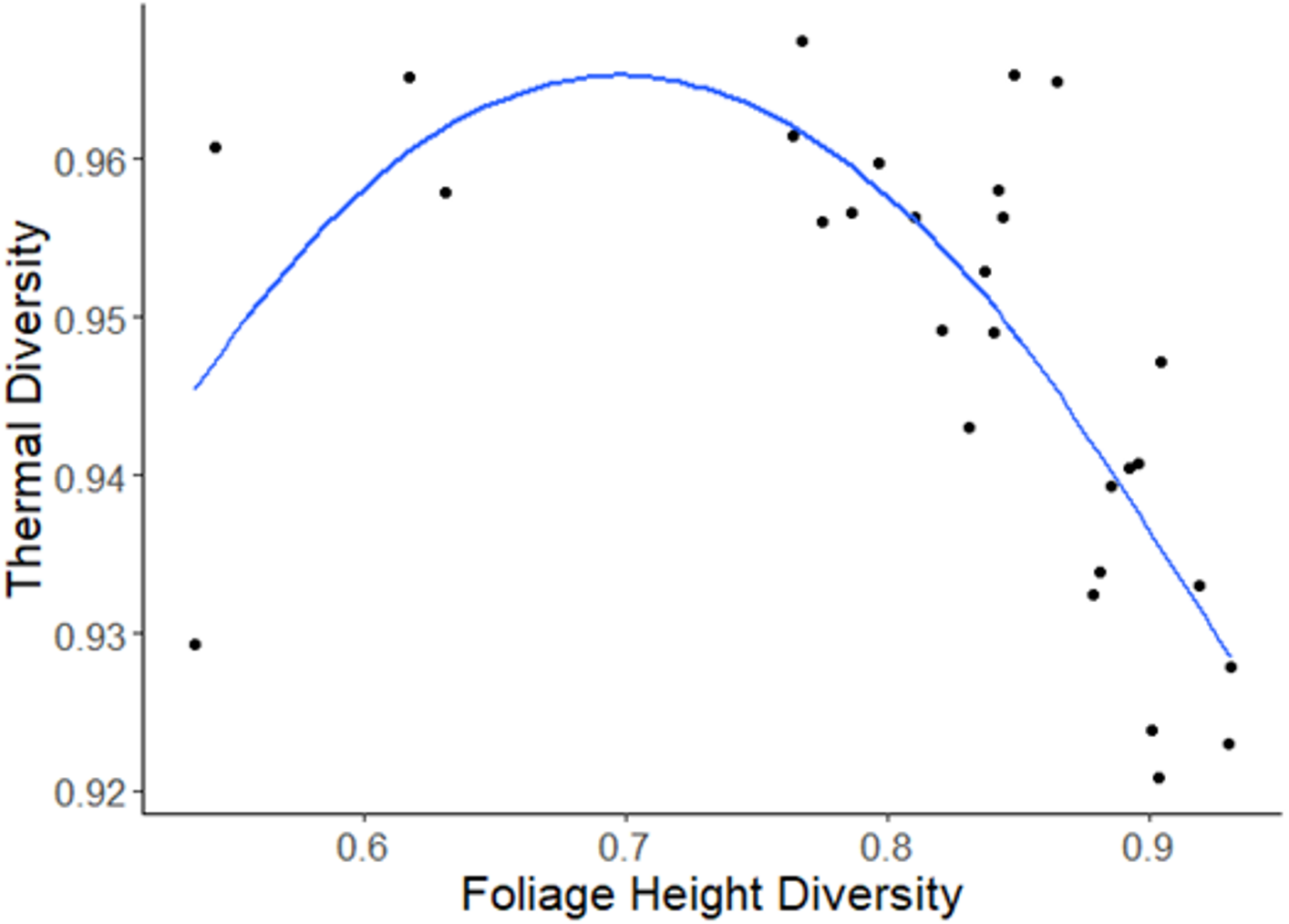
Plot of the relationship between the log of foliage height diversity and thermal diversity. Each point represents one UAV survey.

Foliage height diversity (which was log-transformed) exhibited a peaked relationship with thermal diversity (*R*^2^ = 0.1138, F(1,27) = 4.595, *p* = 0.04123; Fig. 4). Visual inspection indicated that model residuals were normally distributed and homoscedastic.

**Figure 5:**
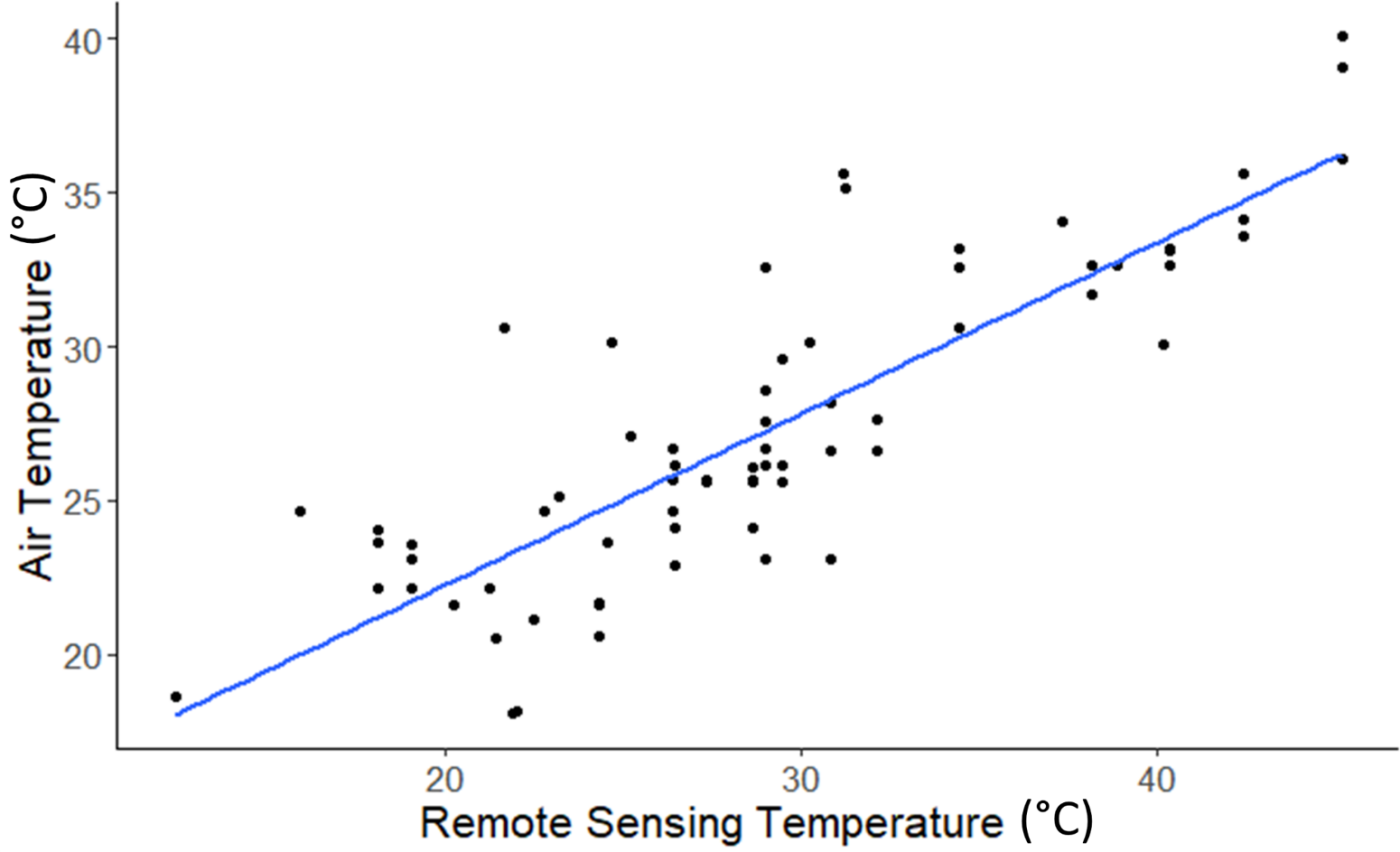
Relationship between remote sensing temperature and air temperature. Remote sensing temperature was extracted from emissivity-corrected remote sensing temperature maps. Air temperature was extracted from *in situ* iButton temperature loggers launched in the study sites.

Air temperature was measured at four different heights with iButton temperature loggers (0 m, 0.05 m, 0.75 m, 1.5 m) and related to air temperature measurements using an ANCOVA. Temperature measurements did not differ statistically within this height range, so all air temperature measurements, regardless of height, were pooled for calibration and validation of remotely sensed soil surface temperature. Air temperature, as measured using *in situ* iButton instruments, related strongly to UAV-based remotely sensed temperatures (*R*^2^ = 0.7129, F(1,72) = 182.2, *p* <  < 10^−4^). Therefore, we used the resulting regression equation, *y* = 0.5558x + 11.12999, to calibrate air temperature values and map them (Fig. 5).

**Figure 6:**
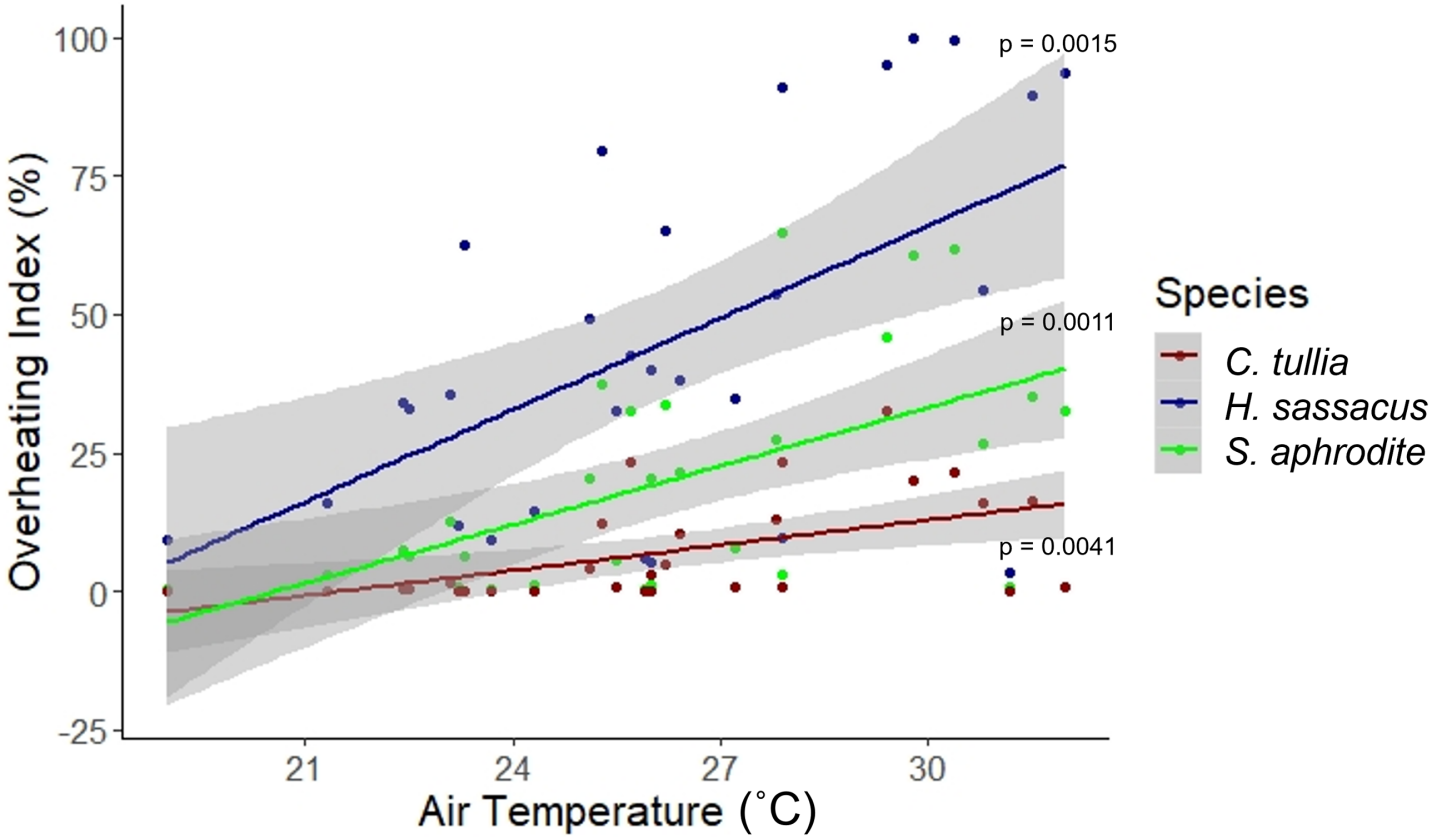
Plot of the overheating index of *H. sassacus*, *S. aphrodite*, and *C. tullia* in relation to coarse air temperature. Coarse air temperature was measured using a handheld humidity meter at the time of each UAV survey.

Coarse air temperature was a significant predictor of the overheating index for *H. sassacus* (*R*^2^ = 0.2902, F(1,27) = 12.45, *p* = 0.0015), *S. aphrodite* (*R*^2^ = 0.3058, F(1,27) = 13.34, *p* = 0.0011), and *C. tullia* (*R*^2^ = 0.2396, F(1,27) = 9.825, *p* = 0.0041). The overheating index position of our three example species diverged increasingly with increasing coarse air temperature (Fig. 6). Handheld humidity meter observations (which measure temperature and humidity) collected *in situ* were assumed to be a validated method of capturing locality-specific air temperature data, while drone-based temperature measurements provide the basis for the site-level metric of overheating and spatial heterogeneity in thermal position. Site level average overheating potential for these species relates to contemporary *in situ* temperature measurements (Fig. 6). These *in situ* values are on the x axis as thermometer measurements of temperature should have very small errors relative to any other technique we employed, including remote sensing measures. Overheating indices for each species were not statistically related to thermal diversity or foliage height diversity.

## Discussion

Here, we demonstrate the feasibility of direct, synoptic measurements of seasonal temperature extremes relative to individual species tolerances using a UAV-borne thermal sensor. Landscape heterogeneity relates strongly to variation in temperature extremes within habitats, relative to the limits of species’ thermal tolerances (see also Carroll et al., 2016; Milling et al., 2018; Suggitt et al., 2018) that are known to affect insect species persistence at broader spatial extents (Soroye, Newbold & Kerr, 2020; Kerr, 2020; Outhwaite, McCann & Newbold, 2022). The method developed here complements temperature measurements that can be interpolated from coarse resolution remote sensing and from meteorological station data ((Kearney et al., 2020; Maclean & Klinges, 2021); Fig. 4). While previous work demonstrates that some insect species’ extinction risks depend on the frequency and intensity of temperature extremes, as measured using the thermal position index (Soroye, Newbold & Kerr, 2020) or derivatives (Outhwaite, McCann & Newbold, 2022), this is the first demonstration that these metrics can be assessed using remote sensing methods within individual habitats.

The importance of microclimatic variation and microclimatic refugia in protecting species from the growing risks of extreme weather has been demonstrated empirically (Bladon et al., 2020; Milling et al., 2018; Riddell et al., 2021). The foundations of such work rely on observed habitat characteristics (Bladon et al., 2020) and frequently employ coarse resolution remote sensing imagery (Riddell et al., 2021) to estimate landscape heterogeneity relative to species’ habitat use. Those techniques are essential for ongoing assessments of microclimatic refugia within habitats because they can provide broad coverage relative to higher resolution, but relatively localized, UAV-based measurements. Nevertheless, more detailed remote sensing at very high resolution (in this study, 5 cm), provides accurate temperature measurements that demonstrate the extent and magnitude of thermal refugia that result from physical heterogeneity within particular habitat patches. These measurements are consistent with observations made at much broader spatial scales (Carroll et al., 2016; Suggitt et al., 2018), though previous work has not assessed microclimatic variation in the context of thermal position. As all survey sites were within the same landscape, and most were very similar in their landscape features, landscape heterogeneity results were very similar. Repeating this methodology in more varied physical landscapes and ecosystems would likely produce more diverse results (Carroll et al., 2016; Gies et al., 2007). We believe the approach we have described here represents a step toward assessing fine-grained thermal constraints in real-world habitats.

The overheating indices for the three study species (*H. sassacus*, *S. aphrodite*, and *C. tullia*; Fig. 2) highlighted the relative impact of localized temperature extremes on individual species relative to species’ thermal limits. Variance in the within-habitat overheating index increased as temperatures rose for each of the three species for which thermal position index (and its spatial average, the overheating index) was measured, suggesting that microclimates persisted in these areas through the warmest periods we observed. As these microclimates depended on structural habitat heterogeneity (*e.g.*, partial canopy cover and shrubs, for example), maintenance of these habitat characteristics and potentially the restoration or addition of those characteristics to habitats could improve species’ resilience to warming conditions, even through the hottest periods observed within this region. Additional work is needed to assess how individual species’ movements and persistence within and among these habitats might relate to thermal conditions, independent from other landscape characteristics, such as habitat connectivity.

Infrared imagery has frequently been used to study surface temperatures in agricultural and geological studies (Faye et al., 2016; Harvey, Rowland & Luketina, 2016; Maes & Steppe, 2019; Sener et al., 2019). However, in ecological studies, air temperature is the primary metric for many species, including adult butterflies. Transformation of UAV-acquired soil surface temperature measurements into air temperature measurements is necessary to transform these remote sensing tools’ outputs into measurements that have the greatest biological relevance for organisms, like butterflies, that spend relatively little time on exposed ground (though, we note that many butterfly species sometimes obtain nutrients from moisture on soil surfaces). Butterfly species are more likely to be vulnerable to surface temperature extremes during egg and larval phases of development (Pincebourde, Dillon & Woods, 2021). It is likely to be possible to alter the framework we have applied here to measure temperatures most relevant to butterflies during those life stages, but more work would be needed to understand temperature variability within the areas through which caterpillars moved as well as on the temperature dependence of ovipositioning behaviour of adult butterflies. Because *in situ* air temperature measurements matched remote sensing metrics quite closely (Fig. 5), we expect that UAV-based thermal measurements, especially if related to thermal tolerances of eggs and larvae, could inform risks of extreme temperatures for butterflies during these earlier life stages.

We found that air temperatures showed little variation from ground level to a height of 1.5 metres within the alvar habitats where we collected *in situ* temperature values. Our measurements were made over areas with vegetated ground cover, which might have reduced temperature variability over this small range. Limestone pavement surface temperatures can be extremely hot in this habitat. Our results would have differed had our ground surface temperatures focused on those areas. Butterflies were not observed to settle onto such surfaces during hot periods. Different habitat types may exhibit other relationships between ground and air temperatures than that observed here, depending on vegetation type, vegetation density, and solar radiance (Gies et al., 2007). Our results suggest species that must engage in behavioural or physiological thermoregulation in hot conditions may face challenges escaping extreme heat by moving upward along vegetated surfaces or adjusting flight heights during foraging. Instead, such species (including the study species) will likely need to rely on heterogeneity within the habitat to find localities where vegetation creates cooler temperatures from ground to canopy and to adjust their activity periods away from the hottest times of day. Disturbances in these habitats that create more homogeneous conditions, such as removing small patches of trees or shrubs, or perhaps even mowing, may eliminate critical thermal microrefugia (Larsen, 2012), and reduce the likelihood of species’ persistence. We predict such effects to become more pronounced as extreme temperatures become more frequent and severe. Remote sensing-based measurements of temperatures within particular habitats will be more relevant and reliable for conservation applications if calibrated by *in situ* temperature measurements. Calibration is necessary as UAV-based estimates of temperature, though strong (*R*^2^ = 0.7129), tended to be slightly lower than *in situ* iButton measurements, perhaps owing to UAV thermal measurements integrating more variable air temperatures above ground level.

Estimates of the thermal position index focused on peak flight seasons for three butterfly species with divergent thermal tolerances. A more thorough estimate of the effects of temperature extremes on butterfly, or other species’, biology would require temperature monitoring throughout the year. We do not discount the potential importance of microclimates at other times of year, but our main focus was on measuring thermal position of habitats during the warmest periods of butterfly activity. Consequently, repeated surveys at each site assessed different temperature regimes, separated by several weeks, which we treated as independent data points. Growing frequency and severity of extreme weather is expected to cause negative population growth among many species, but local losses of species might require several years of such climate-driven declines.

## Conclusions

Monitoring the biological impacts of extreme weather will require a broad array of remote sensing tools and techniques, ranging from broad-scale models drawing on coarse resolution remote sensing to UAV-based measurements that can directly observe within-habitat variation at scales relevant to site-level habitat management. Exposure to extreme temperatures that exceed species’ tolerances increase their extinction risk across broad regions. This study demonstrates that such models can be translated to within-habitat scales, and identify microclimatic variability that is validated by *in situ* temperature measurements for individual species. We believe this work offers one avenue to expand monitoring efforts for biological diversity that can inform practical conservation management.

##  Supplemental Information

10.7717/peerj.13911/supp-1 Supplemental Information 1 Survey information and corresponding environmental conditions and ecological metrics

10.7717/peerj.13911/supp-2 Supplemental Information 2 Air temperature data nearest UAV survey time and corresponding remote sensing temperature data extracted from the remote sensing maps

## Additional Information and Declarations

### Field Study Permissions

The following information was supplied relating to field study approvals (i.e., approving body and any reference numbers): Field experiments and drone use were approved by Ontario Parks.

### Data Availability

The following information was supplied regarding data availability: Raw data are available in the Supplemental Files.
